# Whole genome sequence of *Lactiplantibacillus plantarum* MC5 and comparative analysis of *eps* gene clusters

**DOI:** 10.3389/fmicb.2023.1146566

**Published:** 2023-05-02

**Authors:** Xuefang Zhao, Qi Liang, Xuemei Song, Yan Zhang

**Affiliations:** Functional Dairy Products Engineering Laboratory of Gansu Province, College of Food Science and Engineering, Gansu Agricultural University, Lanzhou, China

**Keywords:** comparative genomics, bioinformatics analysis, carbohydrate metabolic capacity, sugar nucleotide-forming pathways, *eps* gene cluster

## Abstract

**Introduction:**

Probiotic *Lactiplantibacillus plantarum* MC5 produces large amounts of exopolysaccharides (EPS), and its use as a compound fermentor can greatly improve the quality of fermented milk.

**Methods:**

To gain insight into the genomic characteristics of probiotic MC5 and reveal the relationship between its EPS biosynthetic phenotype and genotype, we analyzed the carbohydrate metabolic capacity, nucleotide sugar formation pathways, and EPS biosynthesis-related gene clusters of strain MC5 based on its whole genome sequence. Finally, we performed validation tests on the monosaccharides and disaccharides that strain MC5 may metabolize.

**Results:**

Genomic analysis showed that MC5 has seven nucleotide sugar biosynthesis pathways and 11 sugar-specific phosphate transport systems, suggesting that the strain can metabolize mannose, fructose, sucrose, cellobiose, glucose, lactose, and galactose. Validation results showed that strain MC5 can metabolize these seven sugars and produce significant amounts of EPS (> 250 mg/L). In addition, strain MC5 possesses two typical *eps* biosynthesis gene clusters, which include the conserved genes *epsABCDE*, *wzx*, and *wzy*, six key genes for polysaccharide biosynthesis, and one MC5-specific *epsG* gene.

**Discussion:**

These insights into the mechanism of EPS-MC5 biosynthesis can be used to promote the production of EPS through genetic engineering.

## 1. Introduction

Probiotics are a diverse group of microorganisms widely distributed in nature and an important component of the indigenous microbiota of healthy humans and animals, where they regulate host gut flora (Siezen et al., 2010; Kimoto-Nira, 2018). Strains of *Lactiplantibacillus plantarum* are on the list of probiotic strains available for food and exhibit various probiotic functions such as exopolysaccharide (EPS) production, and antioxidant and antibacterial properties (Peng et al., 2022). EPSs are macromolecular secondary metabolites produced during the growth of lactic acid bacteria (LAB). EPSs produced by LAB are usually divided into homotypic exopolysaccharides (HoPS) and heterotypic exopolysaccharides (HePS) according to their structure (Prete et al., 2021). HoPS are composed of one monosaccharide, whereas HePS are composed of a variety of monosaccharides comprising three to eight repeat units (Feng et al., 2021). EPS-producing LAB are more tolerant to adverse external environments than other LAB and have become the dominant strains during LAB evolution (Fukao et al., 2019a). As EPS produced by LAB can be used as a natural antioxidant, thickener, and stabilizer, EPS-producing LAB (*Streptococcus salivarius* subsp. *thermophilus*, *Lactobacillus delbrueckii* subsp. *bulgaricus*, *Lactococcus lactis* subsp. *lactis*, etc.) are widely used in the dairy industry (Şanlier et al., 2019). In addition, EPS-LAB are reported to play an important role in immune stimulation, anti-inflammation, and cholesterol-lowering (Werning et al., 2022).

Since the first whole-genome sequence of LAB (*Lactobacillus lactis* subsp. *lactis* IL1403) was generated in 2001, whole-genome sequencing (WGS) technology has been applied to find correlations between genetic characteristics of LAB and their functions (Huang et al., 2021). The genomic sequences of many probiotic LAB strains are publicly available, and their essential characteristics have been defined using genomic approaches (Panthee et al., 2019). Qureshi et al. (2020) used WGS to identify genes associated with probiotic properties of *Lactobacillus paracasei* ZFM54 and validated them *in vitro*. Yi et al. (2020) used WGS to predict the genome sequence of *Lp. pentosus* DZ35 and identified two previously unknown genes related to bacteriocins (DZ1 and DZ2). However, few studies have systematically reported on the molecular processes involved in the EPS biosynthesis pathway of LAB. To further develop the EPS-producing capacity of LAB, it is essential to gain insight into the genetics, biosynthetic pathways, regulation, structure, and function of LAB based on known biochemical indicators (Zeidan et al., 2017). The cluster of genes associated with EPS biosynthesis in LAB is known as the *eps* gene cluster (Sun and Zhang, 2021). Typical *eps* gene clusters are 14–25 kb in size and contain less than 30 open reading frames (ORFs). The *eps* gene cluster consists of five highly conserved genes, *epsABCDE*, and a variable region that specifically includes polymerase (*wzy*), flip-flop (*wzx*), genes encoding one or more glycosyltransferases (GTF), and other modifier genes (Zeidan et al., 2017). Song et al. (2018) studied the whole genome sequence of *Lc. casei* LC2W and showed that genes encoding glucose-1-phosphate thymidyltransferase (LC2W_2179), an unidentified EPS biosynthetic protein (LC2W_2188), and an EPS biosynthetic protein (LC2W_2189) were associated with EPS biosynthesis. Xiong et al. (2019) reported that *S. salivarius* subsp. *thermophilus* S-3 has a complete cluster of *eps* genes with 13 ORFs involved in regulation, chain length determination, repeat unit biosynthesis, polymerization, and export during EPS biosynthesis.

High-EPS-producing *Lp. plantarum* MC5, showing good fermentation characteristics and probiotic properties, was isolated from traditional fermented yak yogurt in the Tibetan area of Gansu. Strain MC5 has great application value for production of fermented milk in the Tibetan area and has been used as a compound fermenter for yogurt production (Zhao and Liang, 2022). High EPS production is an important probiotic property of *Lp. plantarum* MC5, and the whole-genome sequence of this strain has been predicted and annotated through various databases (COG, KEGG, GO, CAZy, etc.). In this study, we revealed the general characteristics of the genome and the EPS-producing mechanism of this strain at the genetic level, including the sugar phosphotransferase system (PTS), the *eps* biosynthetic pathways, key enzymes, and gene clusters involved in EPS biosynthesis. The genetic characteristics of strain MC5 are important for the full exploitation and development of its probiotic properties, and also provide a genetic explanation for the high EPS production characteristics of strain MC5.

## 2. Materials and methods

### 2.1. *Lp. plantarum* MC5 culture conditions

*Lp. plantarum* MC5 was isolated from traditional fermented yak yogurt in the Tibetan region of Gansu and stored in skimmed milk glycerol tubes at −80°C. Strain MC5 was inoculated 4% (w/v) in MRS (Solarbio Technology Co., Beijing, China) broth for two generations (34°C, 30 h), and the suspensions were collected by centrifugation (5,000 r/min, 4°C for 10 min) for subsequent experiments.

### 2.2. Extraction of genomic DNA from *Lp. plantarum* MC5

Genomic DNA was extracted from strain MC5 using the CTAB method (cetyltrimethylammonium bromide) (Arseneau et al., 2017). DNA quality control was performed using a NanoDrop 2000 spectrophotometer (Thermo Fisher Scientific, Waltham, MA, USA) to determine DNA concentration, and using a Qubit 3.0 (Life Technologies, Carlsbad, CA, USA) for DNA quantification, followed by 0.8% (w/v) agarose gel electrophoresis to determine the purity of DNA.

### 2.3. Identification and whole-genome sequence analysis of *Lp. plantarum* MC5

#### 2.3.1. Biochemical identification of *Lp. plantarum* MC5

Gram staining was performed using a Gram staining kit (Solarbio Technology Co., Beijing, China). Chemical reagents used for staining included ammonium oxalate crystalline violet staining solution, iodine solution, 95% (v/v) alcohol (decolorization solution), and red staining solution. Under a fluorescence microscope, Gram-positive bacteria appear purple and Gram-negative bacteria appear red (Asuman and Emin, 2018).

To test for catalase activity, A colony of *Lp. plantarum* MC5 growing on MRS agar medium was selected and placed onto a slide; 0.5–1.0 ml 3% (v/v) H_2_O_2_ solution was added slowly, and the production of bubbles within 30 s was observed. Those colonies with bubbles were positive for catalase, and those colonies without bubbles were negative (Asuman and Emin, 2018).

#### 2.3.2. Whole-genome sequence analysis of *Lp. plantarum* MC5

The genome of *Lp. plantarum* MC5 was sequenced using Illumina and PacBio Sequel II platforms (OEbiotech, Qinghai, China). *Denovo* assembly of triple sequencing data was performed using Wtdbg2 software (Ruan and Li, 2019), which obeys the overlap-layout-consensus model. The algorithm was developed based on the Fuzzy Bruin Graph (FBG) theory.

### 2.4. Genome annotation of *Lp. plantarum* MC5

The software, prodigal (full name: Prokaryotic Dynamic Programming Genefinding Algorithm) (Hyatt et al., 2010), was used to predict coding genes. Diamond software (Buchfink et al., 2015) was used to compare predicted coding sequences, taking annotations with e < 1e-5 and filtering for proteins with the highest sequence similarity to obtain functional annotation information. HMMER software (Eddy, 2009) was used to compare these sequences with protein family models to determine the highest-scoring families. The predicted coding sequence regions were then compared against the NR (non-redundant),^1^ KEGG (Kyoto Encyclopedia of Genes and Genomes),^2^ COG (Cluster of Orthologous Groups of proteins),^3^ GO (Gene Ontology)^4^ and CAZy (Carbohydrate-Active Enzymes Database)^5^ databases to complete the basic functional prediction and annotation. Circos (v0.69) software (Krzywinski et al., 2009) was used to present a combination of information in a single genomic circle map.

### 2.5. Comparative genomics analysis of *Lp. plantarum* MC5

The whole-genome sequence of strain MC5 was compared with those of seven strains of LAB (different number of *eps* genes) downloaded from the NCBI database (Table 1).^6^ A whole-genome sequence phylogenetic tree of strain MC5 was reconstructed by Mega 6.4 (Mega Limited, Auckland, New Zealand) using the neighbor-joining method (Davidson and Martín Del Campo, 2020). Protein sequences homologous to the *eps* biosynthesis-related gene cluster in the *Lp. plantarum* MC5 genome were analyzed using blast (see text footnote 6).

**TABLE 1:** Genomic information of other lactic acid bacteria.

Strains	GenBank ID	*eps* genes
*Lactiplantibacillus plantarum* WCFS1	NC_004567.2	*epsG*
*Lactiplantibacillus plantarum* subsp. *plantarum* ST-III	CP002222.1	*eps2ABCE*, *wzx*, *eps3ABDEFHIJ*, *eps4ABCEFGHIJ*
*Lactiplantibacillus plantarum* JDM1	NC_012984.1	*epsD/epsB*, *epsF*
*Lactiplantibacillus plantarum* subsp. *plantarum* NC8	AGRI00000000.1	–
*Lactiplantibacillus plantarum* subsp. *plantarum* ATCC 14917	NZ_ACGZ00000000.2	–
*Lactiplantibacillus plantarum* 19L3	NZ_AWTS00000000.1	–
*Lactiplantibacillus plantarum* strain SMB758	CP118222.1	*epsD/epsB*, *epsF*, *epsG*

### 2.6. Determination of the EPS content of *Lp. plantarum* MC5

*Lp. plantarum* MC5 was inoculated at 4% (v/v) into MRS broth with glucose, lactose, galactose, sucrose, fructose, cellobiose, mannose, trehalose, or arabinose as the sole carbon source, respectively, for 30 h of fermentation. Cultures were then centrifuged (8,000 r/min, 4°C for 20 min), and the supernatant was collected, respectively. The supernatant (5 ml) was mixed with 20% (w/v) trichloroacetic acid (TCA), allowed to stand at 4°C for 24 h, and then centrifuged again (8,000 r/min, 4°C for 20 min). The supernatant was collected, mixed with 95% alcohol (3:1, v/v), and allowed to stand again at 4°C for 24 h. The mixture was centrifuged (8,000 r/min, 4°C for 20 min), and the pellet was suspended in deionized water and dialyzed at 4°C for 2 days using dialysis bags (molecular weights of 8–14 kDa). Afterward, the dialysate was concentrated and vacuum freeze-dried. The EPS concentration in the suspension after dialysis was quantified using the phenol-sulfuric method of Charchoghlyan et al. (2017) and expressed as glucose equivalent with glucose as the standard (Panthavee et al., 2017).

### 2.7. Statistical analysis

All experiments were repeated three times. SPSS 22.0 (Statistical Package for the Social Sciences, Chicago, IL, USA) was used for statistical analysis. Experimental differences were determined using ANOVA and Duncan’s test at 0.05 levels. Figures were drawn using Origin 8.0 software (Statistical Package for the Social Sciences, Northampton, MA, USA).

## 3. Results

### 3.1. Genomic characteristics of *Lp. plantarum* MC5

**FIGURE 1:**
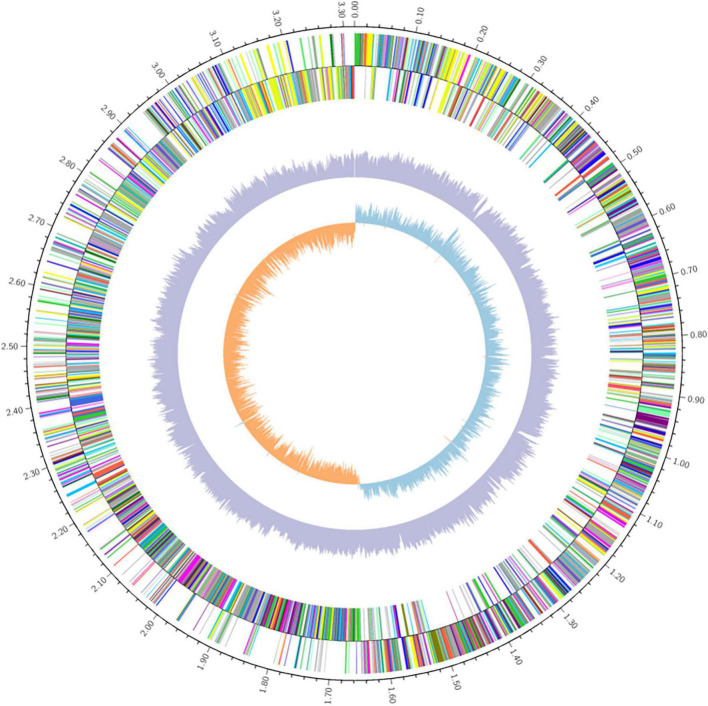
Whole genome circle map of *Lp. plantarum* MC5. From inside to outside: GC skew, GC content, non-coding RNA (rRNA red, tRNA blue, sRNA green), lagging strand COG annotations, leading strand COG annotations.

The whole-genome sequence of *Lp. plantarum* MC5 was obtained using PacBio Sequel II sequencing technology. The genome sequence of MC5 was submitted to NCBI (accession ID: PRJNA897707). The genome of MC5 was 3,317,904 bp in total length, consisting of one circular chromosome of 2,791,014 bp, with a GC content of 45.55%, and two plasmids, plasmid 1 (60,060 bp with 38.79% GC content), and plasmid 2 (15,567 bp with 36.82% GC content). We predicted 3,138 genes, 15 rRNAs (5S, 16S, 23S), 74 tRNAs, and 38 sRNAs (Table 2). The whole-genome circle map of strain MC5 is shown in Figure 1, which comprehensively shows the characteristics of the genome, such as the distribution of genes on the forward and antisense strands, the COG functional classification of genes, GC content, genomic islands, homologous genes, etc.

**TABLE 2:** Assembly statistics of the *Lp. plantarum* MC5 genome.

Sample	Genome size (bp)	Gene number	GC (%)	Gene length/ genome size (%)
Chromosome	2,791,014	3138	45.55	84.12
Plasmid 1	60,060	82	38.79	78.09
Plasmid 2	15,567	46	36.82	56.54

### 3.2. Functional annotation of the *Lp. plantarum* MC5 genome

The predicted protein sequences encoded by the MC5 genome were annotated using NR, KEGG, COG, eggNOG, GO, CAZy, and other databases. If there was more than one annotation result, the one with the best ratio was selected as the gene annotation.

#### 3.2.1. Identification of *Lp. plantarum* MC5

**FIGURE 2:**
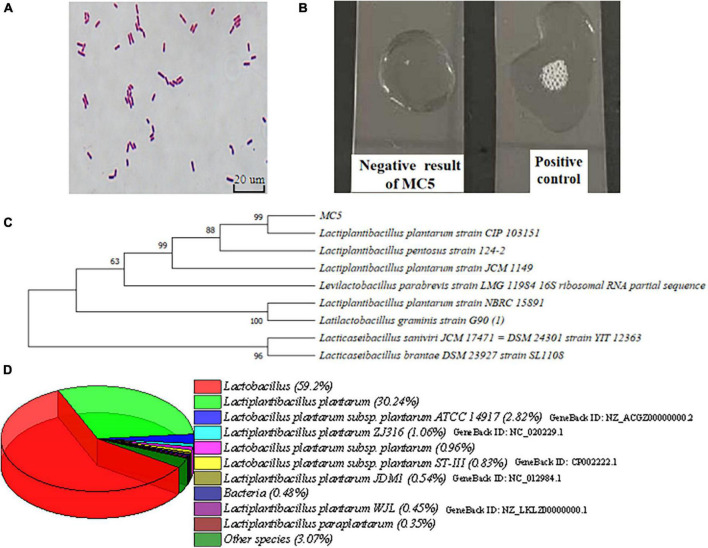
**(A)** Morphology of strain MC5 by fluorescence microscopy; **(B)** catalase test of MC5; **(C)** phylogenetic tree of 16S rRNA gene sequences, and numbers at nodes represent represent homology; and **(D)** species identification of MC5 using annotations from the NR database.

The Gram stain of strain MC5 was purple, and the catalase test did not produce bubbles, indicating that strain MC5 is Gram-positive and catalase-negative. MC5 produced small white colonies on MRS agar and appeared as short rods under fluorescence microscopy (Figures 2A, B). Strain MC5 shared the highest sequence similarity of the 16S rRNA gene with *Lp. plantarum* CIP 103151 (99%) and *Lp. plantarum* JCM 1149 (92%), so strain MC5 was identified as *Lp. plantarum* (Figure 2C, Zhao and Liang, 2023). The NR (non-redundant) database excludes the most significant sources of redundant sequences (EST, STS, GSS, HTGS) and contains protein sequences translated from DNA sequences preserved throughout NCBI GenBank; making the information in this database is very comprehensive. Annotation results from this database contained information on the species annotated according to genes, and statistics on the number of species and genes annotated. Species identification results of MC5 protein sequences in the NR database included *Lactobacillus* (59.20%), *Lp. plantarum* (30.24%), and *Lp. plantarum* ZJ316 (1.06%). NR database annotation of strain MC5 indicated that the strain was most closely related to *Lp. plantarum*, which verified the above identification results (Figure 2D).

#### 3.2.2. KEGG and COG database annotation of *Lp. plantarum* MC5

**FIGURE 3:**
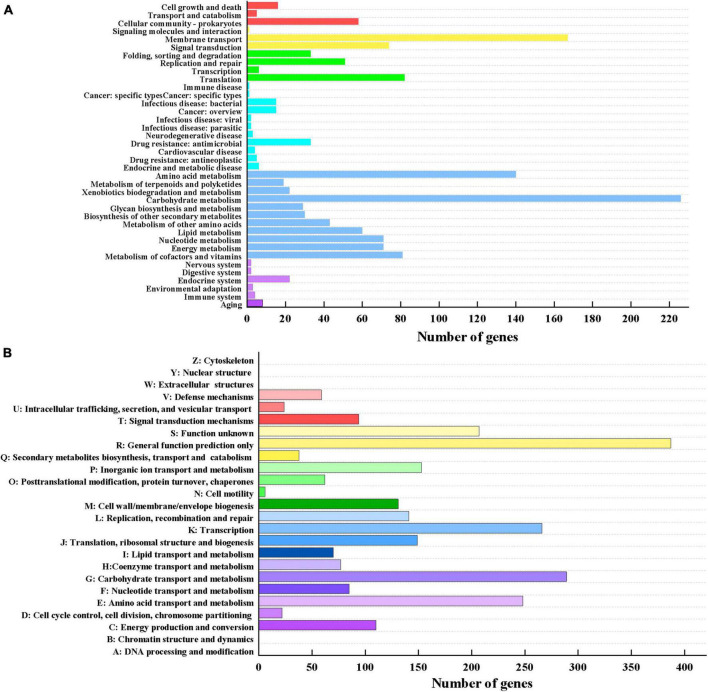
**(A)** KEGG functional annotation of *Lp. plantarum* MC5. The six different colors from top to bottom represent cellular processes, environmental information processing, genetic information processing, human diseases, metabolism, and organismal systems, respectively. **(B)** COG functional annotation of *Lp. plantarum* MC5.

A total of 1,413 genes were annotated in the KEGG database (Figure 3A), classified into six major categories (38 subcategories). There were 79, 242, 172, 87, 792, and 41 annotated genes associated with cellular processes, environmental information processing, genetic information processing, human diseases, metabolism, and organismal system, respectively. The greatest proportion of annotated genes (792) was in the metabolic category, where 226 and 140 genes were related to carbohydrate metabolism and amino acid metabolism, respectively. Genes associated with membrane transport had the highest abundance (167) among genes annotated in the environmental information processing category. Most of the functional genes in the *eps* gene cluster were annotated with membrane transport functions. Genes associated with translation comprised the highest proportion (82) of genes annotated in the genetic information processing category. The above results indicated that MC5 has strong capacity for carbohydrate metabolism, amino acid metabolism, and membrane transportation.

A total of 2,618 functional genes were annotated in the COG database (Figure 3B). Figure 3B shows that a large proportion of proteins were identified in the categories of general function prediction only (R, 387), carbohydrate transport and metabolism (G, 289), transcription (K, 266), and amino acid transport and metabolism (E, 248), while a small proportion were identified in the category of cell motility (N, 6). Notably, among the annotated carbohydrate transport and metabolism genes were a large number of genes related to the biosynthesis of sugar nucleotides, such as those encoding lactose/galactose permease, 1-phosphofructokinase, fructokinase, UDP-glucose-hexose-1-phosphate uridylyltransferase, fructose-bisphosphate aldolase, glucosamine-6-phosphate deaminase, and glucose-6-phosphate-1-dehydrogenase. These results suggested that this strain has a strong capacity for sugar biosynthesis and transport.

### 3.3. CAZy database annotation of *Lp. plantarum* MC5

The above findings revealed that strain MC5 has strong carbohydrate metabolism and EPS biosynthesis potential. To investigate the EPS biosynthesis mechanism, we analyzed the carbohydrate-active enzymes, sugar transport systems, and nucleotide sugar biosynthesis pathways of strain MC5 at the genomic level.

**FIGURE 4:**
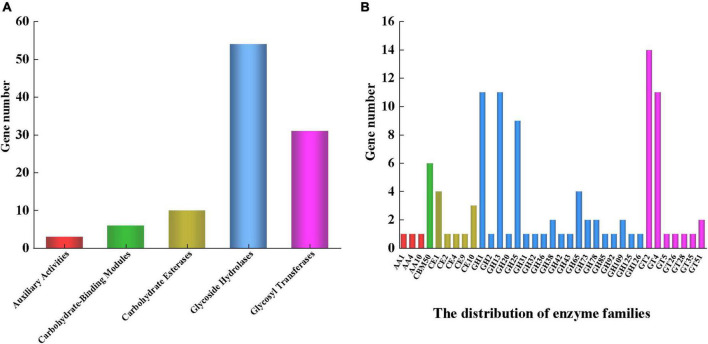
**(A)** CAZy functional annotation of *Lp. plantarum* MC5. **(B)** Distribution of related genes. Red, green, yellow, blue, and purple represent auxiliary activities, carbohydrate-binding modules, carbohydrate esterases, glycoside hydrolases, and glycosyl transferases, respectively.

We identified five major classes of genes for MC5 in the CAZy database, of which glycoside hydrolase (GH, 54) and glycosyltransferase (GTF, 31) related genes were the most abundant (Figure 4A). The most abundant GH genes annotated in strain MC5 were GH1, GH13, and GH25, with 11, 11, and 9, respectively. This could be because the isolation source of strain MC5 (yak yogurt) is rich in carbohydrates. CAZy annotation results showed that GH1 family genes mainly encode beta-glucosidase, beta-galactosidase, and lactase; GH13 family genes mainly encode pullulanase, maltotriose-forming alpha-amylase, and amylosucrase. Thus, the abundance of genomic GH means that strain MC5 can efficiently utilize metabolically different carbon sources, allowing the formation of nucleotide sugar bases. In addition, we annotated in seven classes of GTs family genes in strain MC5, with 14 and 11 genes belonging to GT2 and GT4, respectively (Figure 4B). The above results indicated that strain MC5 has a high capacity for sugar biosynthesis. Notably, the only carbohydrate-binding modules (CBM) family gene annotated in the CAZy results was CBM50 (6), which primarily encodes modules found attached to various enzymes from families GH18, GH19, GH23, GH24, GH25, and GH73, i.e., enzymes cleaving either chitin or peptidoglycan. CBM50 modules are also found in a multitude of other enzymes targeting the peptidoglycan such as peptidases and amidases (Sofie et al., 2021). Ten genes of the carbohydrate esterase (CE) family were annotated (including CE1, CE2, CE9, and CE10), which primarily encode acetyl xylan esterase, diacylglycerol O-acyltransferase, and *N*-acetylglucosamine 6-phosphate deacetylase.

### 3.4. The sugar transport system of *Lp. plantarum* MC5

The annotation results of this experiment showed that strain MC5 has a total of 61 PTS-type sugar transporter proteins associated with a variety of sugars, including 11 sugar-specific transporters such as sucrose PTS EIIBCA or EIIBC components, mannitol PTS EIICBA or EIICB components, cellobiose PTS EIIC component, and fructose PTS EIIBC or EIIC components (Supplementary Table 1). According to the transporter database, these transporters can be classified into thirteen PTS systems with the following distributions: cellobiose PTS (16), beta-glucoside PTS (8), mannose PTS (6), glucitol/sorbitol PTS (4), fructose PTS (3), ascorbate PTS (3), galactitol PTS (3), galactosamine PTS (2), sucrose PTS (2), mannitol PTS (2), sugar PTS (2), *N*-acetylglucosamine PTS (1), and fructoselysine/glucoselysine PTS (1).

The above results indicated that strain MC5 can utilize cellobiose, glucoside, mannose/mannitol, fructose, sucrose, ascorbate, galactitol/galactosamine, glucitol/sorbitol, and *N*-acetylglucosamine. In addition, we found that some of the oligosaccharide phosphate transfer system genes (for cellobiose, mannose, fructose, etc.) in the genome of strain MC5 occurred in multiple copies, and this coding redundancy could enhance the ability of MC5 to utilize these carbon sources. These functional proteins would provide a competitive advantage for the survival of strain MC5 in complex environments.

### 3.5. Nucleotide sugar biosynthesis pathway of *Lp. plantarum* MC5

**FIGURE 5:**
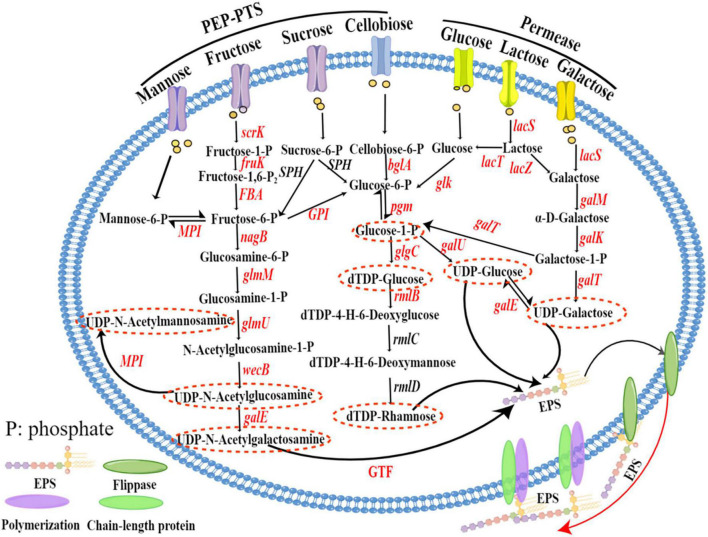
Nucleotide sugar biosynthesis pathways of *Lp. plantarum* MC5. Nucleotide sugar biosynthesis pathways mapped by Figdraw (https://www.figdraw.com/static/index.html#/). Annotated genes are marked in red, and the nucleotide sugars are in red dashed ovals. *scrK*, fructokinase; *SPH*, sucrose-6-phosphate hydrolase; *GPI*, glucose-6-phosphate isomerase; *bglA*, 6-phosphate-β-glucosidase; *pgm*, phosphoglucomutase, *galU*, UTP-glucose-1-phosphate uridylyltransferase; *galE*, UDP-glucose 4-epimerase; *glgC*, glucose-1-phosphate adenylyltransferase; *rmlB*, dTDP-glucose-4,6-dehydratase; *rmlC*, dTDP-4-dehydrorhamnosac-3,5-epimerase; *rmlD*, dTDP-4-dehydrorhamnorea reductase; *glk*, glucokinase; *lacS*, lactose/galactose permease; *lacZ*, β-galactosidase; *galM*, aldose 1-epimerase; *galK*, galactokinase; *galT*, UDP-glucose-hexose-1-phosphate uridylyltransferase; *fruK*, 1-phosphofructokinase; *FBA*, fructose-bisphosphate aldolase; *nagB*, glucosamine-6-phosphate deaminase; *glmM*, phosphoglucosamine mutase; *glmU*, glucosamine-1-phosphate *N*-acetyltransferase; *wecB*, UDP-N-acetylglucosamine 2-epimerase (non-hydrolyzing); *MPI*, mannose-6-phosphate isomerase; GTF, glycosyltransferase.

Nucleotide sugars are precursors in the biosynthesis of EPS. Table 3 shows the key enzymes in EPS biosynthesis annotated in the KEGG database that convert different carbon sources into nucleotide sugars. The sugar sources available to the strain can be inferred from these enzymes. We identified a total of 21 key enzymes involved in the EPS biosynthesis process of strain MC5, involved in the metabolism of mannose, fructose, sucrose, cellobiose, glucose, lactose, and galactose, respectively. The nucleotide sugar biosynthesis pathways of strain MC5 were predicted according to the KEGG metabolic pathways (Figure 5). In LAB, the PTS and permease pathways are essential for the transport of monosaccharides and disaccharides. As shown in Figure 5, mannose, fructose, sucrose, and cellobiose are transported through the PEP-PTS from outside the cell into the cell, where they are phosphorylated to produce mannose 6-phosphate, fructose 1-phosphate, sucrose 6-phosphate, and cellobiose 6-phosphate, respectively. These are then converted to fructose 6-phosphate and glucose 6-phosphate by related enzymes (*fruK*, *FBA*, *GPI*, *bglA*, *SPH*) and are eventually involved in the biosynthesis of UDP-*N*-acetylglucosamine, dTDP-rhamnose, UDP-glucose, and dTDP-glucose. Importantly, glucose 6-phosphate and glucose 1-phosphate are converted by the catalytic reaction of *pgm*, which is involved in the biosynthesis of dTDP-glucose, UDP-glucose, and dTDP-rhamnose. Glucose 1-phosphate is catalyzed by enzymes encoded by *glgC*, *galU*, *galE*, *rmlB*, *rmlC*, and *rmlD* to biosynthesize dTDP-glucose, UDP-glucose, UDP-galactose, and dTDP-rhamnose, respectively. Lactose is converted to glucose 6-phosphate by the catalytic reaction of glucokinase (glk) and 6-phosphate-beta-glycosidase, and is eventually converted to UDP-glucose, dTDP-glucose, and dTDP-rhamnose by the catalytic reactions of glucose-6-phosphate isomerase, UTP-glucose-1-phosphate uridylyltransferase, glucose-1-phosphate adenylyltransferase (*glgC*), dTDP-glucose-4,6-dehydratase (*rmlC*), and dTDP-4-dehydrorhamnorea reductase (*rmlD*).

**TABLE 3:** Key enzymes of the nucleotide sugar biosynthesis process of *Lp. plantarum* MC5 annotated in the KEGG database.

Locus tag	Gene name	Encoded protein
OOD38_0176	*scrK*	Fructokinase
OOD38_2099	*GPI*	Glucose-6-phosphate isomerase
OOD38_2320	*bglA*	6-Phosphate-β-glucosidase
OOD38_0673	*pgm*	Phosphoglucomutase
OOD38_0661	*galU*	UTP-glucose-1-phosphate uridylyltransferase
OOD38_1828	*galE*	UDP-glucose 4-epimerase
OOD38_0020	*glgC*	Glucose-1-phosphate adenylyltransferase
OOD38_1337	*glk*	Glucokinase
OOD38_2959	*lacS*	Lactose/raffinose/galactose permease
OOD38_2973	*lacZ*	Beta-galactosidase
OOD38_2977	*galM*	Aldose 1-epimerase
OOD38_2972	*galK*	Galactokinase
OOD38_2970	*galT*	UDP-glucose–hexose-1-phosphate uridylyltransferase
OOD38_1819	*fruK*	1-phosphofructokinase
OOD38_0304	*FBA*	Fructose-bisphosphate aldolase
OOD38_0210	*nagB*	Glucosamine-6-phosphate deaminase
OOD38_0721	*glmM*	Phosphoglucosamine mutase
OOD38_0422	*glmU*	Glucosamine-1-phosphate N-acetyltransferase
OOD38_1001	*wecB*	UDP-N-acetylglucosamine 2-epimerase
OOD38_2069	*MPI*	Mannose-6-phosphate isomerase
OOD38_1828	*rmlB*	dTDP-glucose-4,6-dehydratase

Therefore, analysis of the predicted genes indicated that strain MC5 can biosynthesize seven sugar nucleotides: UDP-glucose, UDP-galactose, dTDP-glucose, dTDP-rhamnose, UDP-*N*-acetylglucosamine, UDP-*N*-acetylgalactosamine, and UDP-*N*-acetylmannosamine. The genes encoding all of these enzymes were identified in strain MC5, indicating strong tolerance and adhesion of MC5.

### 3.6. Comparative genomic analysis of different *eps* gene strains

**FIGURE 6:**
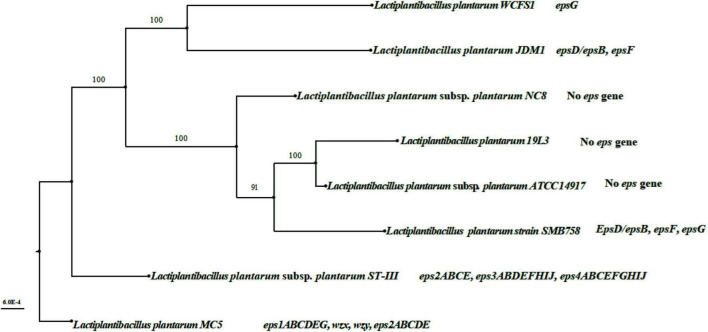
Whole-genome phylogenetic tree of *Lp. plantarum* strains with different *eps* genes. The whole genome sequence of the 7 strains of LAB in Figure 6 was downloaded from the NCBI database, and the GenBank ID of these 7 strains were shown in Table 1. Mega 6.4 (Mega Limited, Auckland, New Zealand) software done their whole genome phylogenetic tree.

Sequences of seven *Lactiplantibacillus* strains containing different numbers of *eps* genes were downloaded from the NCBI database; the names, GenBank IDs, and *eps* genes of these seven strains are shown in Table 1 and Supplementary Table 2. Strains *Lp. plantarum* subsp. *plantarum* NC8, *Lp. plantarum* 19L3, and *Lp. plantarum* subsp. *plantarum* ATCC 14917 had no *eps* genes, strains *Lp. plantarum* WCFS1 and *Lp. plantarum* JDM1 contained very few *eps* genes (*epsB*/*epsD*, *epsG*), and strain *Lp. plantarum* SMB758 contained *epsB*/*epsD*, *epsF*, and *epsG*. Notably, strain *Lp. plantarum* subsp. *plantarum* ST-III contained three *eps* gene clusters, *eps2ABCEFGHI*, *eps3ABDEFHIJ*, and *eps4ABCEFGHIJ*, respectively (Table 1). Figure 6 compares the clustering relationships of LAB containing different numbers of *eps* genes. The three strains without *eps* genes (*Lp. plantarum* subsp. *plantarum* NC8, *Lp. plantarum* 19L3, and *Lp. plantarum* subsp. *plantarum* ATCC 14917) were clustered together, the two strains with few *eps* genes (*Lp. plantarum* WCFS1 and *Lp. plantarum* JDM1) were clustered together, and the strain with a complete *eps* gene cluster (*Lp. plantarum* subsp. *plantarum* ST-III) formed a separate branch. The strains with different numbers of *eps* genes were sister branches of each other. *Lp. plantarum* MC5 and *Lp. plantarum* subsp. *plantarum* ST-III were the closest relatives, as these two strains contained the most *eps* gene clusters and had the highest EPS production capacity.

### 3.7. Comparative analysis of *eps* gene clusters of different lactic acid bacteria

**FIGURE 7:**
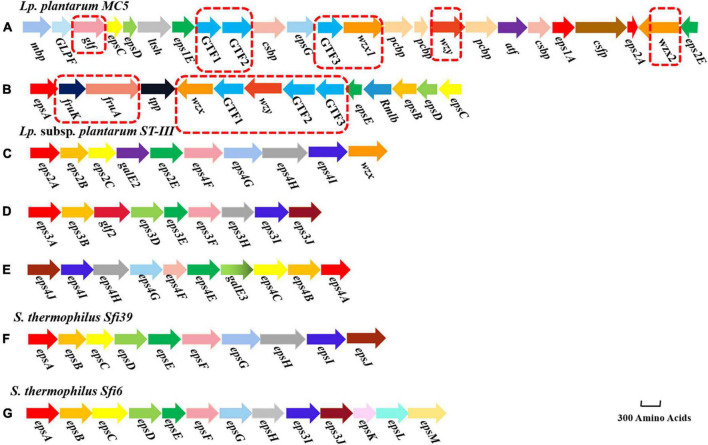
Structure of the *eps* gene clusters of *Lp. plantarum* MC5 (**A**: *eps* cluster 1, **B**: *eps* cluster 2), *Lp. plantarum* subsp. *plantarum* ST-III (**C**: *eps* cluster 1, **D**: *eps* cluster 2, and **E**: *eps* cluster 3), *S. salivarius* subsp. *thermophilus* Sfi39 (**F**: *eps* cluster 1), and *S. salivarius* subsp. *thermophilus* Sfi6 (**G**: *eps* cluster 1). Different colors indicate the different functions of genes. Genes in red dashed boxes are MC5-specific genes.

It has been shown that there is a cluster of related genes located on plasmids or chromosomes that control extracellular polysaccharide biosynthesis; these are organized into four parts: a gene regulatory region (*epsAB*), genes encoding a related protein involved in the detection of polymeric chain length (*epsCD*), genes encoding a glycosyltransferase (GTF) that biosynthesizes extracellular polysaccharide repeat units, and genes for polysaccharide polymerization, transport, and export functions (*wzx* and *wzy*) (Soumya and Nampoothiri, 2021). *Lp. plantarum* subsp. *plantarum* ST-III (GenBank ID: CP002222.1), *S. salivarius* subsp. *thermophilus* Sfi39 (GenBank ID: AF373595.1), and *S. salivarius* subsp. *thermophilus* Sfi6 (GenBank ID: U40830.1) were used as controls for comparing known *eps* gene clusters with those of strain MC5 (Figure 7). Figure 6 shows that genes encoding *eps* in LAB include *epsA*, *epsB*, *epsC*, *epsD*, *epsE*, *epsF*, *epsG*, *epsH*, *epsI*, *glf*, *GTF*, *galE*, *wzx*, and *wzy*. The genes *epsA*, *epsB*, *epsC*, *epsD*, and *epsE* were common among the seven *eps* gene clusters (a-g), indicating that they are highly conserved and stable in the *eps* gene clusters of LAB. However, *epsF*, *epsG*, *epsH*, *epsI*, *epsJ*, and *epsK* were unique to *S. salivarius* subsp. *thermophilus* and encode mainly glycosyltransferases and membrane transfer-associated proteins in this species. The genes *fruK*, *fruA*, *glf*, *GTF*, *wzx*, and *wzy* were unique to *Lp. plantarum* MC5. Glycosyltransferase genes differed in type, number, location, and order among the seven *eps* gene clusters (a-g), making them specific and variable.

Whole-genome analysis of *Lp. plantarum* MC5 showed that its chromosome encodes two complete *eps* genes clusters, *eps* C1 and *eps* C2 (Figures 7A, B). The two *eps* gene clusters were 26 and 14 kb in length and consisted of 24 and 14 ORFs, respectively (Table 4). These two *eps* genes clusters contained *epsABCDE*. The *epsCD* in *eps* cluster 1 was located in the conserved region at the beginning of the cluster and was mainly involved in chain-length regulation of EPS biosynthesis. The first gene in the cluster, *epsA*, has homology to the family of transcriptional regulators, in *Streptococcus pneumoniae* deletion of a similar gene cpsIaA caused a reduction in capsule production (Dertli et al., 2016). The amino acid sequence identity of *eps1A* in *eps* C1 with GenBank ID: MCG0826270.1 was 99.60%, and *eps2A* in *eps* C1 with GenBank ID: WP_151502160.1 was 99.32%, respectively. The amino acid sequence homology of the *epsA* gene of *S. salivarius* subsp. *thermophilus* Sfi39 with ID: MCE2268317.1 was 99.38%. The amino acid sequence homology of the *epsA* gene of *S. salivarius* subsp. *thermophilus* Sfi6 with ID: MCE2192772.1 was 98.35%. This suggested that *eps1A* and *eps2A* have been relatively conserved during evolution. Because the *epsA* gene is mainly responsible for the transcription and regulation of EPS genes during EPS biosynthesis, it determines the direction and degree of expression of *eps* genes. Interestingly, *eps* cluster 1 contained two *epsE* genes, indicating that strain MC5 has a strong ability to start glycosyl transfer. The amino acid sequence identity of *eps1E* and *eps2E* encoded by *eps* C1 was 60.16% (GenBank ID: KRN47847.1) and 100.00% (GenBank ID: ACT61928.1), respectively (Table 4). Although these two *epsA* genes were located at the end of cluster 1, they play a major role in the transcriptional regulation of EPS biosynthesis. In addition, *eps* cluster 1 contained four conserved polysaccharide biosynthesis proteins (amino acid sequence identity of both genes is > 90%) and two capsular biosynthesis proteins that may play a key role in the biosynthesis of EPS. Both proteins were also annotated in strain *Lactobacillus plantarum* subsp. *plantarum* ST-III (GenBank ID: CP002222.1). Furthermore, strain MC5 *eps* C1 contained a unique *epsG* gene that encodes a membrane transporter protein. By contrast, the conserved gene in *eps* C2, located at the beginning of the cluster, was *epsA*, while *epsBCDE* was located at the end of the cluster and had a negative transcriptional orientation. In *eps* C2, the amino acid sequence identity of *epsA* with GenBank ID: OEZ35589.1 was 99.60%, the amino acid sequence identity of *epsB* with GenBank ID: MCG0716518.1 was 99.63%, the amino acid sequence identity of *epsC* with GenBank ID: MCG0585072.1 was 97.70%, the amino acid sequence identity of *epsD* with GenBank ID: WP_213474994.1 was 99.57%, and the amino acid sequence identity of *epsE* with GenBank ID: ADN98952.1 was 100.00%, respectively (Table 4).

**TABLE 4:** Information about the *eps* gene clusters and homologous sequences of *Lp. plantarum* MC5.

Locus tag	Sequence length of AA	Gene name	Gene direction	Gene function	GenBank ID	Homology (%)
***eps* genes cluster 1**						
OOD38_1002	309	*mbp*	+	Integral membrane protein	MCG0749228.1	99.68
OOD38_1003	238	*GLPF*	+	Glycerol uptake facilitator protein	KRO27638.1	96.49
OOD38_1004	372	*glf*	+	UDP-galactopyranose mutase	WP_063490764.1	99.73
OOD38_1005	217	*epsC*	+	Polysaccharide biosynthesis protein	MCG0678676.1	37.91
OOD38_1006	194	*epsD*	+	*CpsD* family tyrosine-protein kinase	WP_181589845.1	31.34
OOD38_1007	371	*ltsh*	+	Class A beta-lactamase-related serine hydrolase	MCT3250941.1	99.73
OOD38_1008	244	*eps1E*	+	Lipopolysaccharide biosynthesis glycosyltransferase	KRN47847.1	60.16
OOD38_1009	298	*GTF1*	+	Glycosyltransferase	WP_195631017.1	99.66
OOD38_1010	354	*GTF2*	+	Glycosyltransferase	WP_063490758.1	99.72
OOD38_1011	332	*csbp*	+	Capsular biosynthesis protein	MPQ37623.1	55.45
OOD38_1012	365	*epsG*	+	Transmembrane protein *epsG*	MBP5842394.1	99.45
OOD38_1013	297	*GTF3*	+	Glycosyltransferase	AUV72009.1	44.37
OOD38_1014	473	*wzx1*	+	Flippase	WP_260203456.1	78.30
OOD38_1015	295	*pcbp*	+	Polysaccharide biosynthesis protein	WP_237421427.1	99.66
OOD38_1016	207	*pcbp*	+	Polysaccharide biosynthesis protein	WP_262339904.1	99.52
OOD38_1017	398	*wzy*	+	Polysaccharide polymerase	ERO40262.1	99.50
OOD38_1018	369	*pcbp*	+	Polysaccharide biosynthesis protein	WP_088467704.1	99.19
OOD38_1019	359	*atf*	+	O-acetyltransferase	MCG0909952.1	99.72
OOD38_1020	258	*csbp*	+	Capsular biosynthesis protein	KRK22728.1	86.67
OOD38_1021	248	*eps1A*	−	Transcription regulator, AraC family	MCG0826270.1	99.60
OOD38_1022	1133	*csfp*	+	Cell surface protein precursor	KRL33646.1	93.88
OOD38_1023	146	*eps2A*	+	MarR family transcriptional regulator	WP_151502160.1	99.32
OOD38_1024	473	*wzx2*	−	Flippase	WP_087613142.1	99.79
OOD38_1025	222	*eps2E*	−	Priming glycosyltransferase	ACT61928.1	100.00
***eps* genes cluster 2**						
OOD38_1818	251	*epsA*	+	DeoR/GlpR transcriptional regulator	OEZ35589.1	99.60
OOD38_1819	305	*fruK*	+	1-phosphofructokinase	WP_003640776.1	100.00
OOD38_1820	655	*fruA*	+	Fructose PTS, EIIABC	WP_128536963.1	99.85
OOD38_1821	440	*Tpp*	+	Serine/threonine protein phosphatase family protein	ETF10433.1	99.55
OOD38_1822	483	*wzx*	−	Oligosaccharide flippase family protein	WP_061871654.1	100.00
OOD38_1823	322	GTF1	−	Glycosyltransferase	WP_133279738.1	99.69
OOD38_1824	424	*wzy*	−	Polysaccharide polymerase	WP_128468359.1	99.76
OOD38_1825	342	GTF2	−	Glycosyltransferase	WP_112297297.1	99.71
OOD38_1826	363	GTF3	−	Glycosyltransferase	WP_003640783.1	99.72
OOD38_1827	221	*epsE*	−	Priming glycosyltransferase	ADN98952.1	100.00
OOD38_1828	313	*rmlB*	−	dTDP-glucose 4 6-dehydratase	KZU50552.1	99.25
OOD38_1829	273	*epsB*	−	Tyrosine protein phosphatase	MCG0716518.1	99.63
OOD38_1830	235	*epsD*	−	*CpsD* family tyrosine-protein kinase	WP_213474994.1	99.57
OOD38_1831	252	*epsC*	−	Tyrosine-protein kinase transmembrane modulator *epsC*	MCG0585072.1	97.70

Glycosyltransferases (GTFs) can transfer different nucleotide sugars, and GTF and EPS molecular diversity are closely related. Strain MC5 *eps* C1 and C2 had abundant genes encoding GTFs (6), indicating a high polymorphism of GTFs. We also found that the *GTF* genes of MC5 mainly encoded galactosyltransferase, glucosyltransferase, and D-mannosaminyltransferase, leading to the assumption that this strain can biosynthesize EPS of different structures. In *eps* C1 the amino acid sequence identity of GTF1 with GenBank ID: WP_195631017.1 was 99.66%, the amino acid sequence identity of GTF2 with GenBank ID: WP_063490758.1 was 99.72%, and the amino acid sequence identity of GTF3 with GenBank ID: AUV72009.1 was 44.37%, respectively (Table 4). In *eps* C2, the amino acid sequence homology of GTF1 with GenBank ID: WP_133279738.1 was 99.69%, the amino acid sequence homology of GTF2 with GenBank ID: WP_112297297.1 was 99.71%, and the amino acid sequence homology of GTF3 with GenBank ID: WP_003640783.1 was 99.72%, respectively (Table 4). In addition, the *GTF* genes included genes encoding furanyltransferase, mannosyltransferase, and α-glucosyltransferase. Both *eps* C1 and C2 of strain MC5 contained genes encoding *wzx* and *wzy*, located in the middle region of the cluster (both of which have > 90% amino acid sequence identity, Table 4). Notably, *eps* C1 encoded two *wzx* genes, indicating that cluster 1 has a strong repeat unit transport capacity. The above findings suggested that strain MC5 has a unique EPS biosynthesis mechanism and a strong EPS production capacity.

### 3.8. Validation of the EPS-producing capacity of *Lp. plantarum* MC5

Sugar source utilization capacity of MC5 and the number of key enzymes that metabolize the sugar were predicted from KEGG database annotation results (Table 5). MC5 was predicted to contain key enzymes for metabolism of nine sugar sources (glucose, lactose, sucrose, arabinose, trehalose, fructose, cellobiose, galactose, and mannose), indicating that strain MC5 can metabolize these nine sugar sources. However, key metabolic enzymes of five sugar sources (mannitol, xylose, raffinose, rhamnose, and sorbitol) were not predicted, indicating that strain MC5 cannot metabolize these five sugars. We predicted two permeases for raffinose metabolism and three PTS transport systems for sorbitol metabolism, indicating that these two sugars might enter the cells of strain MC5 but not be metabolized.

**TABLE 5:** Prediction of sugar source utilization capacity of MC5 and number of key enzymes that metabolize the sugar based on KEGG database annotation results (number of enzymes).

Items	Glucose	Trehalose	Lactose	Mannitol	Sucrose	Arabinose	Xylose
Number of enzymes	6	3	9	0	4	2	0
Result	+	+	+	−	+	+	−
Items	Raffinose	Fructose	Cellobiose	Rhamnose	Sorbitol	Galactose	Mannose
Number of enzymes	2 permeases	8	4	0	3 PTS	8	6
Result	−	+	+	−	−	+	+

“+” means that the sugar can be utilized; “−” means that the sugar cannot be utilized.

**FIGURE 8:**
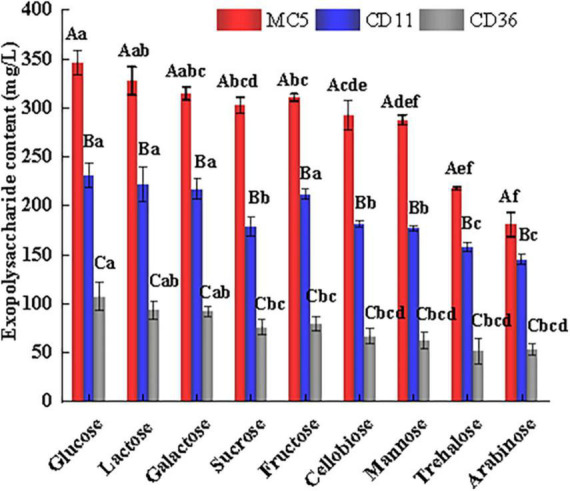
Sugar metabolism types and EPS production of *Lp. plantarum* MC5 (red column). Experiments were repeated three times. *Lp. plantarum* CD11 and *Lp. plantarum* CD36 (blue and gray columns) were the control groups for moderate EPS production and low EPS production, respectively. Capital letters (A, B, and C), respectively, represent the differences in EPS produced by different strains using the same carbon source (*p* < 0.05). Lowercase letters (a, b, c, d, e, and f) represent the differences between EPS produced by the same strain using different carbon sources (*p* < 0.05). ANOVA tests were used to determine significant differences between treatments with a significance level of *p* < 0.05 by the SPSS 22.0 package program. Error bars represent the standard errors (SE) of the mean value (*n* = 3), and the experiment was repeated three times.

Based on the predicted nucleotide sugar biosynthesis pathways of strain MC5 at the gene level, we verified the sugar metabolism capacity of MC5 and the corresponding EPS yield (Figure 8). Using *Lp. plantarum* CD11 and *Lp. plantarum* CD36 as controls, we predicted that these three strains of *Lp. plantarum* can metabolize glucose, lactose, galactose, sucrose, fructose, cellobiose, mannose, trehalose, and arabinose. However, the EPS yield of MC5 was significantly higher than that of CD11 and CD36, which was consistent with the predicted results of the PTS transport systems and sugar metabolism pathways above (sections “3.4. The sugar transport system of *Lp. plantarum* MC5 and 3.5. Nucleotide sugar biosynthesis pathway of *Lp. plantarum* MC5”). Furthermore, when these nine sugars were used as the sole carbon source for the biosynthesis of EPS, the EPS yields of the seven sugars predicted by the genomic sequence were all greater than 250 mg/L, indicating that all these sugars are involved in the biosynthesis of EPS. The highest EPS yields were achieved when glucose and lactose were the only carbon sources, at 345.72 and 327.30 mg/L. Although only some of the enzymes of the trehalose and arabinose metabolic pathways were identified in the genome prediction results, the validation results showed that strain MC5 could metabolize both sugars normally, mainly due to the complexity of the EPS biosynthesis pathways and the possible existence of other gene loci and enzymes involved in the metabolism of trehalose and arabinose. The high EPS yield of strain MC5 results from it having two *eps* gene clusters and the highest number of predicted genes related to carbohydrate metabolism (226) in the KEGG database. The above results showed that strain MC5 had a strong capacity for sugar metabolism and can produce large amounts of EPS.

In addition, all three strains produced the highest amount of EPS when glucose and lactose were used as carbon sources and the lowest amount when trehalose and arabinose were used as carbon sources, which may be because all three strains were isolated from a dairy source (traditional fermented yak milk in Gansu) and have undergone evolutionary adaptation to dairy polysaccharides. Among them, *Lp. plantarum* CD36 produced the lowest EPS content, probably due to the low expression of the *eps* gene cluster in this strain.

## 4. Discussion

We studied the genomic features of MC5 and the biosynthetic pathways of EPS from both genomic and phenotypic aspects. Whole-genome sequence results indicated that probiotic MC5 has a strong metabolism of carbohydrates and amino acids. Genes identified in MC5 are chiefly involved in the metabolism of sucrose, galactose, fructose, mannose, and starch, and some intermediates of their metabolic pathways may be involved in the formation of nucleotide sugars (Xiong et al., 2019). MC5 can grow and reproduce on MRS agar containing glucose, sucrose, fructose, or lactose, respectively, validating the annotation results from the KEGG database. Lactic acid bacteria are reported to be rich in carbohydrate metabolism genes and can use a variety of carbohydrates, which might also provide energy for growth (Ghattargi et al., 2018). Our results indicate that strain MC5 has a strong capacity for sugar biosynthesis and transport. Huang et al. (2021) reported that strains with many genes related to amino acid and carbohydrate transporter metabolism (*Lp. plantarum* DMDL 9010) are highly resistant to the outside environment and have good proliferation capacity, supporting the results of this study. Pessione (2012) also reported that some genes related to carbohydrate metabolism and amino acid decarboxylation play an important regulatory role for LAB in the host gut.

CAZy annotation results showed that GH1 family genes mainly encode beta-glucosidase, beta-galactosidase, and lactase; GH13 family genes mainly encode pullulanase, maltotriose-forming alpha-amylase, and amylosucrase. Lammens et al. (2009) reported that the GH family of genes plays an important role in a variety of biological processes including sugar biosynthesis, cellular metabolism, and signaling. Graebin et al. (2016) and Liang et al. (2022) reported that the glucosidases encoded by GH1 and GH13 family genes produce monosaccharides by hydrolyzing glucosidic bonds, providing precursors for the biosynthesis of EPS. The main component of EPS (nucleotide sugars) might be an important control factor for EPS production levels; this has been evaluated extensively in *Lactobacillus* cells that produced EPS by regulating nucleotide sugar biosynthetic pathways (Kleerebezem and Hugenholtz, 2003). The abundance of genomic GH in the genome of strain MC5 suggests that it can efficiently utilize different carbon sources, thus allowing the formation of nucleotide sugar bases. In addition, the GT family is one of the key gene families for EPS biosynthesis, but there is a high variability of GT family genes in EPS-producing LAB (Zeidan et al., 2017). GT family genes can catalyze the formation of various sugar conjugates such as oligosaccharides and polysaccharides, as well as the biosynthesis of biologically important carbohydrates (Ibrahim et al., 2016). GT2 and GT4 enzymes are associated with the biosynthesis of sugars such as sucrose, cellulose, lipopolysaccharides, and chitosan, which (especially polysaccharide compounds) may be related to the final viscosity, water-holding capacity, and other qualities of fermented milk (Oehme et al., 2019). The above results indicate that strain MC5 has a high capacity for sugar biosynthesis. Our results also show that strain MC5 used as a compound starter can largely improve the EPS content and rheological properties of yogurt (Zhao and Liang, 2022), verifying the functions of genes predicted in strain MC5.

Genomic analysis showed that MC5 has 11 sugar-specific PTS and seven nucleotide sugar biosynthesis pathways. The PTS system is one of the major functional components of the intracellular transport of glycosides (Morabbi Heravi and Altenbuchner, 2018). It generally consists of the non-specific energy-coupled protease (EI) and phosphocarrier protein (HPr) and the sugar-specific PTS transporter protease complex (EII). The histidine residue at position 15 of the HPr protein is a phosphorylation site dependent on phosphoenolpyruvate, which is catalyzed by EI to form His-P-HPr and functions as a transmitting phosphate group during sugar uptake (Liu et al., 2019). Fagbemigun et al. (2021) reported that four strains have mannose PTS transport systems, with two strains having additional glucitol/sorbitol PTS transport systems, after analyzing their whole-genome sequences, and found that these two strains had greater sugar utilization capacity. Sugar-specific PTS analysis indicated that strain MC5 can utilize cellobiose, glucoside, mannose/mannitol, fructose, sucrose, ascorbate, galactitol/galactosamine, glucitol/sorbitol, and *N*-acetylglucosamine.

Endogenous levels of the major component of EPS, nucleotide sugars, can be an important control on the level of EPS production (Hugenholtz et al., 2000; Kleerebezem and Hugenholtz, 2003), which has been evaluated extensively in *Lactobacillus*. Hamon et al. (2014) identified the gene encoding *rmlB* (Locus tag: Gene-1828) as being associated with low pH tolerance in *Lp. plantarum*. Adhesion to the epithelium is crucial for a probiotic strain, and genes encoding lactate dehydrogenase (7, Locus tags: Gene-0322, 0469, 0765, 0944, 1038, 1733, 2041) and 6-phosphogluconate dehydrogenase (2, Locus tags: Gene-1043, 1310) have been identified, which are known to promote bacterial adhesion to mucin and epithelial cells (Izquierdo et al., 2009). Previous studies have identified glutathione peroxidase (Locus tag: Gene-0206) as playing an important role in the acid stress response of LAB (Lee et al., 2010). In addition, Fukao et al. (2019b) reported that EPS consisting of glucose and *N*-acetylglucosamine in *Levilactobacillus brevis* KB290 is associated with its wrinkled colony morphology, suggesting that EPS is responsible for the formation of wrinkled colonies. Although the presence of some precursor biosynthetic genes in the *eps* cluster indicates the presence of this sugar in EPS, the deletion of this gene cannot be taken as an indicator of the absence of this sugar in EPS (Dimopoulou et al., 2014). Therefore, it is necessary to use WGS techniques to reveal the relationship between the genotypic and phenotypic profiles of probiotics.

In addition, the EPS biosynthesis capacity of *Lactobacillus* is controlled by a cluster of *eps* genes located on chromosomes or plasmids (Deo et al., 2019). Strain MC5 has two complete *eps* biosynthesis gene clusters (*epsABCDE*, *wzx*, *wzy*, *epsG*). These two *eps* genes clusters contain *epsABCDE* (key genes for EPS biosynthesis) (Kanmani et al., 2018). The expression of *epsCD* regulatory modules is reported to be coordinated in LAB (Deo et al., 2019). The most critical gene in EPS biosynthesis is *epsE* (priming glycosyltransferase), which primarily encodes a membrane-associated glycosyltransferase that cannot catalyze the attachment of glycosidic bonds but can transfer 1-phospho-glucose to an undecaprenylphosphate lipid carrier on the cytoplasmic surface of the membrane (Horn et al., 2013). Previous studies have reported that *epsA* is required for the attachment of EPS to the cell wall (Chan et al., 2014). In addition, *eps* cluster 1 contains six conserved EPS biosynthetic proteins (> 90% homology) that may play a key role in the biosynthesis of EPS (Song et al., 2018); however, their specific role needs to be further investigated. Kanmani et al. (2018) and Deo et al. (2019) also reported *eps* genes clusters, *wzx*, *wzy*, and *GTF* in LAB, and the structure of the conserved genes (*epsABCDE*) in strain MC5 was similar to that of other EPS-producing LAB, suggesting that the prediction was reliable. The *wzx* and *wzy* genes are mainly involved in glycosyltransport and polysaccharide polymerization in EPS biosynthesis (Xu et al., 2019). In *L. lactis* subsp. *cremoris* SMQ-461, genes with the same function as the *wzy* polymerase and *wzx* flippase genes are named *epsH* and *epsM*, respectively (Dabour and LaPointe, 2005). Zeidan et al. (2017) reported LAB containing genes associated with EPS production that are capable of producing biofilms and attaching to mucosal surfaces. Martino et al. (2016) compared the *eps* gene clusters of several *Lp. plantarum* strains and showed that they were highly variable, while both *eps* gene clusters of strain MC5 have a highly conserved phenotype. This difference may be caused by the different isolation sources of these *Lp. plantarum* strains and the sequencing technology employed.

Previous studies also reported monosaccharides and oligosaccharides that can be widely utilized by LAB (Hugenholtz et al., 2000). Our sugar metabolism verification showed that MC5 is able to metabolize the seven sugars (mannose, fructose, sucrose, cellobiose, glucose, lactose, and galactose) predicted by the genomic sequence and is able to produce significant amounts of EPS. Notably, MC5 could additionally metabolize trehalose and arabinose, but EPS production was lower than those of the other seven sugars, probably due to lower transcript levels of the relevant genes when MC5 metabolized these two sugars. Xiong et al. (2019) also confirmed the ability of *S. salivarius* subsp. *thermophilus* S-3 containing the *eps* gene cluster to metabolize lactose, galactose, and glucose. In addition, genes for the uptake and utilization of glucan, lactose, and galactose and the biosynthesis of glucan also have been identified in the genome of *L. delbrueckii* TUA4408L (Kanmani et al., 2018), providing support for the results of this study. The above results provide better insight into the mechanism of EPS biosynthesis at the levels of biosynthesis pathways, gene clusters, and EPS production.

## Data availability statement

The datasets presented in this study can be found in online repositories. The names of the repository/repositories and accession number(s) can be found below: NCBI, PRJNA897707.

## Author contributions

XZ: conceptualization, methodology, validation, formal analysis, data curation, writing—original draft preparation, and writing—editing. QL: conceptualization, methodology, investigation, resources, writing—review and editing, supervision, and funding acquisition. XS: writing—review and editing, supervision, and funding acquisition. YZ: conceptualization, supervision, and funding acquisition. All authors have read and agreed to the published version of the manuscript.

## Footnotes

1
https://www.ncbi.nlm.nih.gov/

2
http://www.genome.jp/kegg/pathway.html

3
https://www.ncbi.nlm.nih.gov/COG/

4
http://geneontology.org/

5
http://www.cazy.org/

6
https://blast.ncbi.nlm.nih.gov/Blast.cgi
