# Supplementary material for: Whole genome sequence of Lactiplantibacillus plantarum MC5 and comparative analysis of eps gene clusters
Source: Front Microbiol. 2023 May 2;14:1146566. doi: 10.3389/fmicb.2023.1146566 (PMC10185785; doi:10.3389/fmicb.2023.1146566)
Supplement: Supplementary file 1 [file Table_1.docx]

Supplementary Material

Whole genome sequence and *eps* gene clusters comparative analysis of

*Lactiplantibacillus plantarum MC5*

Xuefang Zhao*, Xuemei Song, Yan Zhang

*** Correspondence:** Qi Liang: liangqi@gsau.edu.cn

# Supplementary Table

**Supplementary Table 1.** Sugar-specific phosphate transport system in *Lp. plantarum MC5*.

| Locus tag | Sequence length of AA | Description | Pathway definition |
| --- | --- | --- | --- |
| OOD38_0177  OOD38_2713 | 651  641 | sucrose PTS system EIIBCA or EIIBC component [EC:2.7.1.211] | Starch and sucrose metabolism |
| OOD38_0215 | 612 | mannitol PTS system EIICBA or EIICB component [EC:2.7.1.197] | Fructose and mannose metabolism |
| OOD38_0217 | 149 | mannitol PTS system EIIA component [EC:2.7.1.197] | Fructose and mannose metabolism |
| OOD38_0228  OOD38_0261  OOD38_0393  OOD38_0396  OOD38_0985  OOD38_0994  OOD38_1214  OOD38_2514  OOD38_2615  OOD38_2996 | 419  441  453  452  438  435  451  108  452  445 | cellobiose PTS system EIIC component | Starch and sucrose metabolism |
| OOD38_0244  OOD38_0245  OOD38_0774  OOD38_2644  OOD38_2731  OOD38_3001  OOD38_3005 | 665  653  483  617  664  624  665 | beta-glucoside PTS system EIICBA component [EC:2.7.1.-] | Phosphotransferase system (PTS) |
| OOD38_0505 | 324 | mannose PTS system EIIAB component [EC:2.7.1.191] | Fructose and mannose metabolism |
| OOD38_0506  OOD38_2028 | 268  265 | mannose PTS system EIIC component | Fructose and mannose metabolism |
| OOD38_0507  OOD38_2027 | 310  282 | mannose PTS system EIID component | Fructose and mannose metabolism |
| OOD38_0510 | 139 | mannose PTS system EIIA component [EC:2.7.1.191] | Fructose and mannose metabolism |
| OOD38_0511  OOD38_2726 | 172  106 | fructose PTS system EIIB component [EC:2.7.1.202] | Fructose and mannose metabolism |
| OOD38_0772  OOD38_2513 | 163  148 | sugar PTS system EIIA component [EC:2.7.1.-] | Gluconeogenesis / Starch and sucrose metabolism (PTS) |
| OOD38_0819  OOD38_1212  OOD38_2322 | 110  112  115 | cellobiose PTS system EIIA component [EC:2.7.1.196 2.7.1.205] | Starch and sucrose metabolism |
| OOD38_1060 | 88 | phosphocarrier protein HPr | Phosphotransferase system (PTS) |
| OOD38_1061 | 576 | phosphoenolpyruvate-protein phosphotransferase (PTS system enzyme I) [EC:2.7.3.9] | Phosphotransferase system (PTS) |
| OOD38_1213  OOD38_2323  OOD38_2514 | 99  105  108 | cellobiose PTS system EIIB component [EC:2.7.1.196 2.7.1.205] | Starch and sucrose metabolism |
| OOD38_1819 | 305 | 1-phosphofructokinase [EC:2.7.1.56] | Fructose and mannose metabolism (PTS) |
| OOD38_1820  OOD38_2725 | 655  366 | fructose PTS system EIIBC or EIIC component [EC:2.7.1.202] | Fructose and mannose metabolism |
| OOD38_1922  OOD38_3107 | 119  125 | glucitol/sorbitol PTS system EIIA component [EC:2.7.1.198] | Fructose and mannose metabolism |
| OOD38_2026 | 138 | fructoselysine/glucoselysine PTS system EIIA component [EC:2.7.1.-] | Phosphotransferase system (PTS) |
| OOD38_2128 | 662 | N-acetylglucosamine PTS system EIICBA or EIICB component [EC:2.7.1.193] | Amino sugar and nucleotide sugar metabolism |
| OOD38_2211 | 140 | N-acetylgalactosamine PTS system EIIA component [EC:2.7.1.-] | Galactose metabolism |
| OOD38_2212 | 271 | galactosamine PTS system EIID component | Galactose metabolism |
| OOD38_2213 | 269 | galactosamine PTS system EIIC component | Galactose metabolism |
| OOD38_2214 | 160 | galactosamine PTS system EIIB component [EC:2.7.1.-] | Galactose metabolism |
| OOD38_2450 | 177 | beta-glucoside PTS system EIIA component [EC:2.7.1.-] | Phosphotransferase system (PTS) |
| OOD38_2482 | 660 | N-acetylglucosamine PTS system EIICBA or EIICB component [EC:2.7.1.193] | Amino sugar and nucleotide sugar metabolism |
| OOD38_2727 | 153 | fructose PTS system EIIA component [EC:2.7.1.202] | Fructose and mannose metabolism |
| OOD38_3016 | 456 | ascorbate PTS system EIIC component | Ascorbate and aldarate metabolism |
| OOD38_3017 | 98 | ascorbate PTS system EIIB component [EC:2.7.1.194] | Ascorbate and aldarate metabolism |
| OOD38_3018 | 152 | ascorbate PTS system EIIA or EIIAB component [EC:2.7.1.194] | Ascorbate and aldarate metabolism |
| OOD38_3022 | 423 | galactitol PTS system EIIC component | Galactose metabolism /PTS |
| OOD38_3023 | 98 | galactitol PTS system EIIB component [EC:2.7.1.200] | Galactose metabolism /PTS |
| OOD38_3024 | 156 | galactitol PTS system EIIA component [EC:2.7.1.200] | Galactose metabolism /PTS |
| OOD38_3108 | 339 | glucitol/sorbitol PTS system EIIB component [EC:2.7.1.198] | Fructose and mannose metabolism |
| OOD38_3109 | 183 | glucitol/sorbitol PTS system EIIC component | Fructose and mannose metabolism |

**Supplementary Table 2.** *Eps* gene clusters information of other 7 strains.

| Strains | *Eps* gene name | Protein ID |
| --- | --- | --- |
| *Lp. plantarum* WCFS1 | *epsG* | WP_011101305.1 |
| *Lp. plantarum* subsp*. plantarum* ST-III | *eps2A*  *eps2B*  *eps2C*  *eps2E*  *eps3A*  *eps3B*  *eps3D*  *eps3E*  *eps3F*  *eps3H*  *eps3I*  *eps3J*  *eps4A*  *eps4B*  *eps4C*  *eps4E*  *eps4F*  *eps4G*  *eps4H*  *eps4I*  *eps4J*  *wzx* | ADN98179.1  ADN98180.1  ADN98181.1  ADN98183.1  ADN98203.1  ADN98204.1  ADN98206.1  ADN98207.1  ADN98208.1  ADN98209.1  ADN98210.1  ADN98211.1  ADN98956.1  ADN98955.1  ADN98954.1  ADN98952.1  ADN98184.1  ADN98185.1  ADN98186.1  ADN98187.1  ADN98947.1  ADN98188.1 |
| *Lp. plantarum* JDM1 | *epsD/epsB*  *epsF* | WP_015640554.1  WP_003643865.1 |
| *Lp. plantarum subsp. plantarum* NC8 | *-* | - |
| *Lp. plantarum subsp. plantarum* ATCC 14917 | *-* | - |
| *Lp. plantarum* 19L3 | *-* | - |
| *Lp. plantarum strain* SMB758 | *epsD/epsB*  *epsF*  *epsG* | WDQ20187.1  WDQ21455.1  WDQ21011.1 |
